# Clinical features of cough syncope: a systematic review

**DOI:** 10.1007/s10072-026-09467-6

**Published:** 2026-10-07

**Authors:** Yanqin Fan, Meng Hou, Haili Wang, Jingke Le, Liming Cao

**Affiliations:** 1 Department of Neurology, The First Affiliated Hospital of Shenzhen University, Shenzhen, 518000 China; 2 Department of Neurology, Shenzhen Second People’s Hospital, Shenzhen, China; 3 Kuichong Community Health Service Center, Shenzhen Dapeng New District Medical and Health Group, Shenzhen, China; 4 Guangxi University of Chinese Medicine, Nanning, China; 5 Affiliated Hospital of traditional Chinese medicine, Chongqing Three Gorges Medical College, Chongqing, China; 6 Hunan Provincial Key Laboratory of the Research and Development of Novel Pharmaceutical Preparations, Changsha Medical University, Changsha, China

**Keywords:** Anti-infective therapy, Cough syncope, Pulmonary infection, Reflex syncope, Severe cough

## Abstract

**Background:**

Cough syncope (CS) is a rare subtype of syncope that remains underrepresented in evidence-based medicine. We aimed to characterize the clinical features of CS, with the aim of improving recognition and optimizing management strategies.

**Methods:**

We present five cases of CS alongside a systematic review of its clinical characteristics based on published literature to improve recognition and optimize management. Reports of eligible patients were screened, and demographic, clinical, diagnostic, treatment, and outcome data were analyzed.

**Results:**

This study included 60 patients with a mean age of 56 ± 15 years, 91% of whom were male. The most common underlying triggers were respiratory diseases, including pulmonary infections (35%) and bronchitis (22%). Common comorbidities included hypertension (26%), bronchial asthma (18%), and diabetes mellitus (15%). CS episodes were directly triggered by severe coughing in 93% (26/28) of patients. Episodes occurred at least twice daily in 20% of patients, and limb convulsions accompanied syncope in 22%. Symptoms improved after treatment in 98% of cases. After a median follow-up of 3.75 months (Interquartile Range: 2–12 months), the recurrence rate of syncope was 11%. CS, predominantly triggered by severe coughing, primarily affected middle-aged and older men.

**Conclusions:**

CS predominantly affects middle-aged and older men and is frequently associated with pulmonary infections. Acute pulmonary infections serve as a common underlying trigger for CS, and patients are at risk of recurrent syncope and falls. Disease management should be focused on treating chronic airway disease, particularly chronic obstructive pulmonary disease.

**Supplementary Information:**

The online version contains supplementary material available at https://doi.org/10.1007/s10072-026-09467-6.

## Introduction

Cough syncope (CS) is a rare subtype of syncope characterized by a sudden, transient loss of consciousness brought on by paroxysmal, forceful coughing [1]. It has notable clinical significance because of its sudden onset and complex pathophysiology. However, current evidence remains limited, with most data derived from case reports. The term “cough syncope” was first introduced by Kerr et al. in 1953 [2]. CS accounts for approximately 2.6% of adult syncope cases [3] and predominantly affects middle-aged men and patients with chronic airway diseases [4, 5]. Moreover, CS is frequently misdiagnosed as epilepsy or a cardiogenic event, potentially leading to unnecessary diagnostic testing.

The central pathophysiological mechanism of CS involves a sudden increase in intrathoracic pressure induced by coughing, which reduces venous return, causes an abrupt decline in cardiac output, and results in cerebral hypoperfusion, producing a characteristic hemodynamic “domino effect” [3, 6]. Recent studies have reported that autonomic dysregulation, characterized by enhanced vagal tone and an inadequate sympathetic response, plays a critical role in triggering syncope [7]. In addition, comorbidities, such as pulmonary hypertension and chronic obstructive pulmonary disease (COPD), may further impair cerebral autoregulation and increase susceptibility to CS. Although a close temporal relationship between coughing and syncope is typical, diagnosis relies heavily on detailed history-taking and identification of triggers, because routine investigations (including electrocardiography, electroencephalography, and imaging) are often unremarkable.

Therapeutic strategies are evolving from empirical trigger avoidance toward mechanism-based, targeted interventions, including airway optimization, and autonomic modulation. Nonetheless, because of the rarity of CS, absence of standardized diagnostic criteria, and limited multidisciplinary collaboration, the condition remains underrepresented in evidence-based medicine. In this study, we briefly report five cases and conduct a systematic review of published literature to characterize the clinical features of CS, with the aim of improving recognition and optimizing management strategies.

## Methods

We briefly present five cases of CS diagnosed in our hospital, along with a systematic review of its clinical characteristics based on existing literature.

### Study search strategy and selection criteria

A comprehensive search on PubMed and the China National Knowledge Infrastructure using MeSH terms (“Syncope” and “Cough”) and keywords (“cough syncope,” “cough-induced syncope,” and “cough”) was conducted to identify literature on CS from database inception to December 31, 2025.

The inclusion criteria for the articles were as follows: (1) meeting the diagnostic criteria for CS [1, 8] defined as a transient loss of consciousness immediately after coughing with rapid recovery and exclusion of alternative causes; and (2) detailed case descriptions, including case reports and case series. The exclusion criteria were as follows: (1) reviews, guidelines, or expert consensus without specific CS cases; (2) unavailable full-text articles; and (3) incomplete case records lacking more than one key item, including demographics, comorbidities, syncope description, treatment, outcomes, or relevant test findings.

**Fig. 1:**
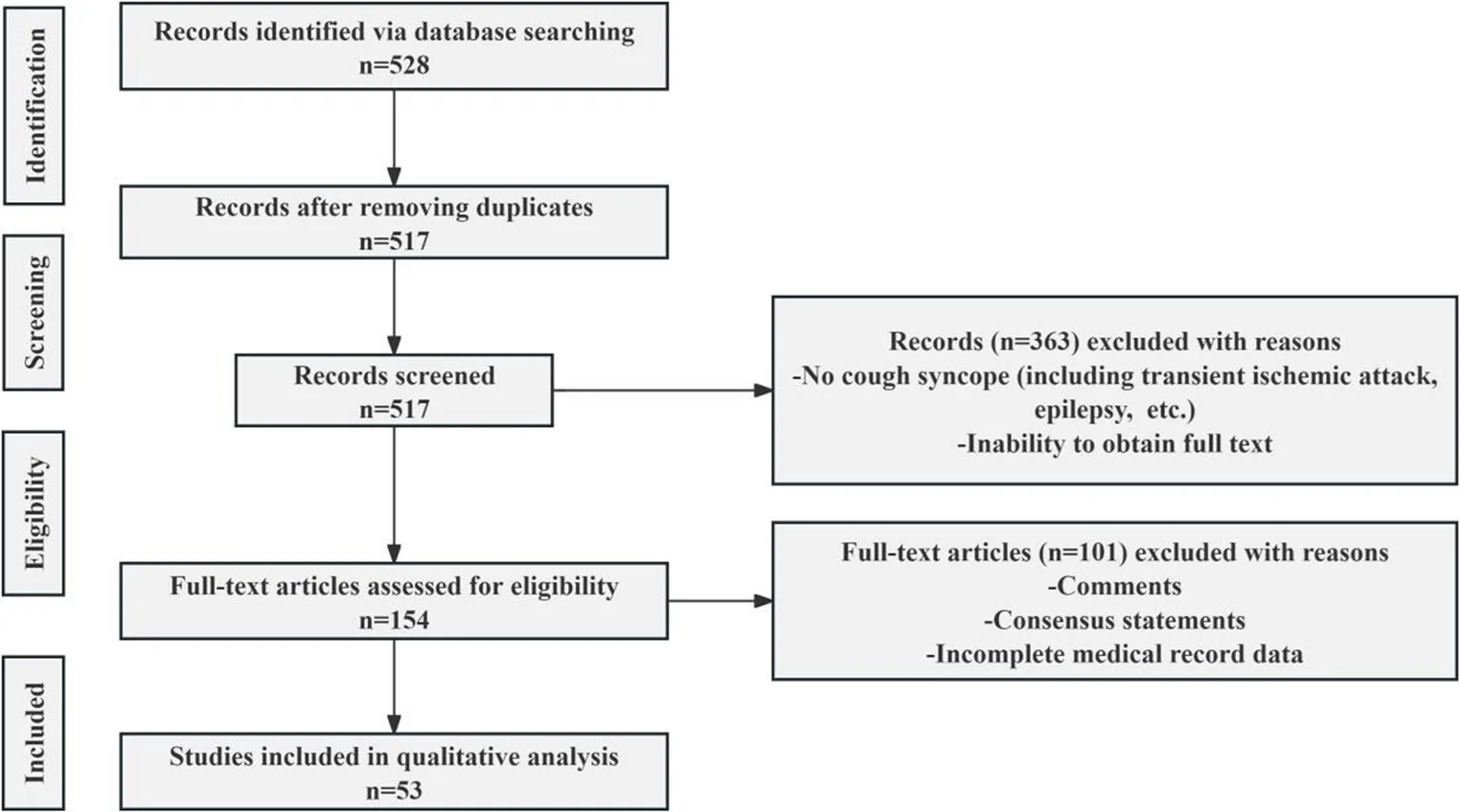
Literature search and screening process

The initial search identified 528 articles. After screening, 60 patients with CS from 53 articles were included. The screening process is shown in Fig. 1.

### Data extraction

Demographic information, comorbidities, chief concerns/main symptoms/positive signs, medical history, main test results, treatment, treatment outcomes, and follow-up data were extracted for cases in the literature [9–61].

## Results

The clinical characteristics of the five patients diagnosed with CS at our hospital (the first five cases in Online Resource Table 1) and the 60 patients with CS identified in the literature review are summarized in Online Resource Table 1. For the five patients diagnosed at our institution, the principal findings were as follows: (1) four patients were young to middle-aged men with overweight (body mass index > 24 kg/m²) and concomitant pulmonary infection; (2) severe coughing was the primary precipitating factor for syncope in all five patients (Online Resource Video 1); (3) frequent CS episodes occurred, with up to ≥ 10 episodes in a single day; (4) the head-up tilt test yielded positive results in four of the five patients; and (5) symptom resolution during follow-up in all patients after active anti-infective and antitussive therapy.

In Table 1, we summarize and analyze data from all patients with CS. Regarding direct triggers, among patients with documented cough severity, 93% (26/28) experienced CS immediately after a bout of severe coughing; whereas 28% (18/65) developed syncope after cold exposure, defined as brief exposure (minutes to hours) to environments significantly colder than body temperature or standard comfort levels. Cold exposure constitutes a key indirect precipitant of CS via provoking pulmonary infection. The most common underlying triggers were respiratory diseases, including pulmonary infections (35%) and bronchitis (22%).

**Table 1:** Demographic and clinical characteristics of 65 patients with cough syncope

Indicator			Frequency or Positive /Total	Percentage
Male sex			59/65	91%
Age, y	< 45		18/65	28%
	45–59		18/65	28%
	≥ 60		29/65	45%
History of smoking			17/65	26%
Overweight (BMI ≥ 24 kg/m^2^)			12/22	55%
Precipitating Factor	Direct triggers of CS	Severe cough	26/28	93%
		Mild cough	2/28	7%
		Cough severity not described	37/65	57%
	Triggers of respiratory tract infections	Cold exposure	18/65	28%
	Underlying triggers	Pulmonary infection	23/65	35%
		Acute/chronic bronchitis	14/65	22%
		Upper respiratory tract infection	7/65	11%
Comorbidities	Hypertension		17/65	26%
	Bronchial asthma		12/65	18%
	Diabetes mellitus		10/65	15%
	Coronary heart disease		8/65	12%
	OSA		8/65	12%
	Stroke		7/65	11%
	GERD		5/65	8%
	Pulmonary tumor		5/65	8%
	Pericarditis/pericardial effusion		4/65	6%
	COPD		3/65	5%
	Pertussis		2/65	3%
	Silicosis		1/65	2%
	Subclavian artery steal syndrome, internal carotid artery and vertebral artery occlusion		1 case each	2%
Auxiliary Examination	Sleep monitoring		6/6	100%
	Chest radiographs/CT		41/54	76%
	Gastroscopy/24-h esophageal pH monitoring		4/7	57%
	Pulmonary function/bronchodilation test		8/15	53%
	Sputum /blood culture		5/10	50%
	Cardiac ultrasound		17/35	49%
	Immunoglobulin E		3/7	43%
	Cranial CT/magnetic resonance imaging		18/44	41%
	Elevated WBC or neutrophil ratio		18/48	38%
	Electrocardiogram		14/48	29%
	Electroencephalography		3/28	11%
Clinical Manifestation	Syncope complicated with convulsion		14/65	22%
	CS attacks ≥ 2 times/day		13/65	20%
	Accompanied by falls with secondary injury		12/65	18%
	Loss of consciousness > 2 min		5/65	8%
Treatment	Anti-infection therapy		42/49	86%
	Antitussive, expectorant, antispasmodic, and anti-asthmatic therapy		51/65	78%
	Enhance gastrointestinal motility or acid suppression		5/5	100%
Follow-up (*n* = 38, a median follow-up of 4 months)				
Syncope Recurrence			4/38	11%

*BMI* body mass index, *COPD* chronic obstructive pulmonary disease, *CS* cough syncope, *CT* computed tomography, *GERD* gastroesophageal reflux disease, *OSA* obstructive sleep apnea, *WBC* white blood cell. Cough intensity was not specified in 37 cases

Regarding comorbidities, hypertension was present in 26%, bronchial asthma in 18%, diabetes mellitus in 15%, and a history of obstructive sleep apnea (OSA) in 12% (8/65) of patients. In addition, 20% (13/65) of patients experienced two or more CS episodes per day, and limb convulsions accompanied syncope in 22% (14/65). Most CS episodes (8 out of 9) occurred while patients were sitting or standing, whereas only 1 out of 9 patients experienced an episode in the supine position.

Laboratory and imaging findings revealed elevated white blood cell counts and neutrophil ratios in 38% (18/48) of patients and increased serum immunoglobulin E levels in 43% (3/7). Chest radiography and computed tomography demonstrated evidence of pulmonary infection in 76% (41/54) of patients. Among those who underwent gastroscopy or 24-h esophageal pH monitoring, reflux esophagitis was diagnosed in 57% (4/7). Electrocardiographic abnormalities were identified in 29% (14/48) of patients, most commonly tachycardia and premature beats. Echocardiographic abnormalities—primarily left ventricular diastolic dysfunction and mild valvular regurgitation—were detected in 49% (17/35).

Overall, symptom improvement was achieved in 98% (64/65) of patients. After a median follow-up of 3.75 months (IQR: 2–12 months), syncope recurred in 11% (4/38) of patients.

## Discussion

In this systematic review, we characterize the clinical manifestations of CS and confirm that middle-aged and older men are the predominant patient population, with pulmonary infection serving as the primary underlying precipitant and cold exposure as the major predisposing factor for respiratory tract infections.

### Predisposing conditions and pathogenesis of CS

We identified severe paroxysmal coughing as the most critical direct trigger of CS, with 93% of patients experiencing syncope immediately after intense coughing episodes. Violent coughing can generate a sharp increase in intrathoracic pressure, which may exceed 300 mmHg—far surpassing the pressure gradient required for normal venous return. This impedes venous blood flow to the right atrium, markedly reducing preload and right ventricular filling, and consequently, decreasing systemic blood pressure [3]. Compared with controls, patients with CS exhibited greater declines in systolic blood pressure and prolonged recovery times during coughing episodes [6]. As left ventricular filling depends on pulmonary return from the right ventricle, impaired right ventricular output diminishes left ventricular preload, ultimately precipitating a sharp drop in systemic cardiac output. Meanwhile, recent evidence increasingly highlights the role of autonomic dysregulation, particularly exaggerated vagal activation (“vagal-cardiac inhibitory reflex”) in susceptible individuals. In susceptible individuals, severe coughing can excessively activate vagal afferent fibers, triggering a “vagal-cardiac inhibitory reflex” that can lead to abrupt bradycardia or, in rare cases, cardiac arrest [6].

Cold exposure was identified as an important indirect predisposing factor, as 28% of patients developed coughing due to respiratory tract infections after cold exposure, which subsequently precipitated syncope. Respiratory tract infections are the most common underlying triggers, including pulmonary infections (35%) and bronchitis (22%). Most patients with CS had underlying chronic conditions, particularly respiratory diseases. Respiratory diseases included pulmonary infection, COPD, bronchitis, bronchial asthma, and OSA. These disorders serve as a pathological basis for coughing and may impair cardiac function. For example, COPD is frequently complicated by pulmonary arterial hypertension, and right ventricular hypertrophy or dilatation can compress the left ventricle, further restricting left ventricular filling [62]. Acute infection acts as a precipitant enabling the hemodynamic cascade, while chronic airway disease (especially COPD) serves as the permissive background. Although pulmonary infection is commonly identified at onset, it often represents an acute exacerbation superimposed on underlying chronic airway disease—particularly COPD—which confers long-term susceptibility to cough-induced hemodynamic compromise.

Common comorbidities in patients with CS included hypertension (26%), bronchial asthma (18%), and diabetes mellitus (15%). These conditions may increase susceptibility to CS by altering autonomic nervous system function, vascular reactivity, and cerebral perfusion regulation. GERD was identified in 8% of patients. GERD-associated cough is typically chronic, paroxysmal, and often exacerbated at night and triggered by meals, with 57% of patients diagnosed with reflux esophagitis or exhibiting abnormal esophageal pH monitoring. Pericardial disease, including pericardial effusion or tamponade, was present in 6% of patients; the proposed mechanism involves mechanical cardiac compression, reduced cardiac output, and exacerbation of pre-existing hemodynamic disturbances by coughing. Echocardiography revealed cardiovascular abnormalities in 49% of patients, indicating impaired cardiac function.

### Clinical characteristics of CS

We found that most patients with CS were middle-aged or older men. This finding aligns with established descriptions in the literature [63], suggesting that age and male sex are important risk factors. Owing to a natural decline in baroreceptor sensitivity, older patients are particularly at risk for CS. In this study, approximately one-quarter of patients had a history of smoking, and over half were overweight or obese. Previous studies have reported associations between CS and factors such as age, male sex, smoking, overweight, OSA, and COPD [64]. Patients with CS are prone to frequent episodes during the acute phase, which can easily lead to falls and subsequent trauma. 20% of patients experienced two or more daily syncopal episodes during the acute phase, indicating a high short-term recurrence risk that warrants clinical attention. Furthermore, 18% of patients experienced falls during syncope, resulting in injuries or accidents. Notably, syncope occurred in three patients while driving, leading to traffic accidents. In combination with the six analogous cases reported previously [65], these observations underscore the serious public safety risks posed by CS episodes. Notably, episode frequency in CS is highly dynamic and closely tied to the underlying triggers—particularly respiratory infections. In the acute phase, such infections may induce clustered, high-frequency episodes of more than ten attacks per day, whereas during recovery or in patients with chronic airway disease, episodes tend to be sporadic and less frequent.

Limb convulsions accompanied syncope in 22% of patients, a rate considerably higher than that (5–10%) reported in patients with vasovagal syncope [1]. Abnormal postures were reported in 42 (65%) and muscle jerks in 33 cases (51%) [66] of syncope. This clinical feature increases the risk of misdiagnosis as epilepsy. Jerks and tonic postures were common in syncope, but semiology differed from convulsive seizures [66]. Syncope-associated limb convulsions may reflect severe cerebral ischemia. Although syncope usually causes unconsciousness for < 2 min, a small subset of patients (8%) in this study experienced unconsciousness lasting > 2 min, suggesting impaired cerebral perfusion recovery. This phenomenon may be related to autonomic nervous system dysfunction due to underlying conditions (e.g., hypertension, diabetes mellitus, or stroke) or reduced cerebrovascular autoregulatory capacity. Such patients represent a “high-risk subgroup” of CS that requires focused clinical attention.

Elevated white blood cell counts or neutrophil ratios were observed in 38% of patients, consistent with infection. Increased immunoglobulin E levels observed in 43% of patients suggested that a subset had atopic diathesis or allergic disease. Chest radiography or computed tomography revealed pulmonary infection in 76% of patients. Abnormal pulmonary function and bronchodilation tests were present in 53%, indicating the central contribution of pulmonary infection to the pathophysiology of CS. Sleep monitoring in six patients with suspected OSA yielded a positive rate of 100%, establishing OSA as an important predisposing factor for CS. OSA contributes to hypertension, progressing to cardiac remodeling and cardiac dysfunction. Among those who underwent gastroscopy or 24-h esophageal pH monitoring, reflux esophagitis was diagnosed in 57% (4/7).

### CS treatment

Anti-infective therapy is an important treatment option for patients with CS and pulmonary infection, with 86% of patients receiving such interventions in this study. Following effective anti-infective therapy combined with antitussive agents, the cough symptoms were significantly controlled, and no recurrent syncope during follow-up in the four patients at our hospital. However, absence of syncope during follow-up does not equate to cure. Timely and adequate anti-infective therapy can directly alleviate airway inflammation and irritation caused by secretions, thereby reducing coughing. Effective anti-infective therapy markedly reduced cough frequency and intensity in patients with acute exacerbations of COPD or bronchial infection, thereby preventing CS episodes [67, 68].

Meanwhile, antitussive, expectorant, antispasmodic, and anti-asthmatic treatments are key components in controlling cough and interrupting the “cough–syncope” vicious cycle; 78% of patients in this study received such regimens. Rational use of antitussive agents is essential for patients with post-infectious cough or severe coughing. Central antitussives reduce the sensitivity of the cough center to suppress the cough reflex, whereas peripheral antitussives decrease the excitability of sensory nerve endings in the airways; both approaches have been proven effective in reducing CS [68]. Additionally, gabapentin acts as an antitussive by inhibiting neurotransmitter release and is an effective treatment option for patients with CS [53].

The clinical features of CS associated with GERD are characterized by the occurrence of syncope, mostly during sleep, which may be accompanied by reflux symptoms, such as acid regurgitation and retrosternal burning pain. Such patients often show little symptomatic improvement with routine anti-infective, antitussive, and anti-asthmatic treatments. In most of these patients, the administration of proton pump inhibitors combined with prokinetic agents results in a significant reduction in cough frequency and intensity, with a corresponding decrease in the number of concurrent syncope episodes. In the present study, five patients [38, 39, 42, 52, 59] with this subtype achieved favorable outcomes with acid-suppressive and gastrointestinal prokinetic treatments. This finding suggests that screening for GERD is important when investigating the etiology of cough-related syncope.

In addition, massive pericardial effusion caused by inflammation or tumors, which induces cardiac tamponade, increases the risk of CS. In this study, four patients [47, 57, 60, 61] who developed CS secondary to pericardial effusion achieved complete resolution after pericardiocentesis and drainage. This result underscores the importance of cardiac structural and functional evaluations in the etiological identification of CS. Therefore, timely and targeted interventions are of paramount importance.

Smoking cessation and weight reduction are important modifiable risk factors—especially given that 26% of patients had a smoking history and > 50% of those with BMI data were overweight in this study. Our study and a previously published cohort of Chinese patients with cough syncope report nearly identical clinical profiles (predominantly middle-aged males, overweight status, and a smoking history) compared with populations reported worldwide [69].For patients with recurrent CS, lifestyle interventions—including smoking cessation and weight optimization—should be integrated into long-term management plans to reduce cough burden and autonomic susceptibility. Furthermore, despite routine counseling on weight management and smoking cessation, long-term adherence to these lifestyle interventions is consistently poor among Chinese patients; this trend is consistent with global patterns. Growing evidence indicates that galvanic vestibular stimulation can modulate a variety of autonomic functions and, under certain pathological conditions, help restore physiological parameters to the normal healthy range [70].

In this study, approximately 10% developed syncope recurrence during follow-up, underscoring the need for long-term monitoring of this patient subset. An earlier study reported a 3-year recurrence rate of 35% for CS, with 82% of recurrences occurring within the first 2 years [71]. Therefore, noting that the recurrence rate of CS remains notably high is essential.

### Limitations

Therapeutic efficacy was summarized from individual case reports, which lacked unified evaluation criteria (such as percentage reduction in cough frequency, degree of decrease in syncope episode frequency, and improvement in quality-of-life scores). Substantial variations in the medication types, dosages, and treatment durations across studies made it difficult to compare the effectiveness of specific treatments. Furthermore, this systematic review is inherently limited by its reliance on previously published case reports, which vary substantially in the completeness of reporting. Key clinical variables, such as body position during syncope, were not systematically documented across the included studies. For example, this variable was reported in only 9 of 65 patients, precluding a meaningful analysis of postural influences on CS occurrence.

## Conclusion

CS predominantly affects middle-aged and older men and is frequently associated with pulmonary infection, GERD, or pericardial effusion. Episodes are typically directly triggered by severe coughing. Some patients may present with convulsions, which can be misdiagnosed as epilepsy, highlighting the importance of careful differential diagnosis. Management depends on accurate etiological identification and targeted therapy, including anti-infective, pericardiocentesis with drainage, acid-suppressive regimens, smoking cessation and weight reduction. however, a significant proportion remain at risk of recurrence and require long-term follow-up. Future research should be focused on establishing multicenter prospective registries, elucidating pathophysiological mechanisms, developing standardized challenge protocols to objectively assess therapeutic effect, and optimizing individualized therapeutic strategies.

## Supplementary Information

Below is the link to the electronic supplementary material.

Supplementary Material 1 (DOCX 56.8 KB)

Supplementary Material 2 (MP4 7.16 MB)

## Data Availability

The datasets used and analyzed during the current study are available from the corresponding author on reasonable request.
