# Supplementary material for: Clinical features of cough syncope: a systematic review
Source: Neurol Sci. 2026 Oct 7;47(11):849. doi: 10.1007/s10072-026-09467-6 (PMC13645981; doi:10.1007/s10072-026-09467-6)
Supplement: Supplementary file 1 [file 10072_2026_9467_MOESM1_ESM.docx]

**Journal: *Neurological Sciences***

**Clinical features of cough syncope: a systematic review**

**Yanqin Fan^1,2 #,^ *, Meng Hou^3^, Haili Wang^4^, Jingke Le^5^, Liming Cao^1,6 #,^***

^1^Department of Neurology, The First Affiliated Hospital of Shenzhen University, Shenzhen, China

^2^Department of Neurology, Shenzhen Second People's Hospital, Shenzhen, China

^3^Kuichong Community Health Service Center, Shenzhen Dapeng New District Medical and Health Group, Shenzhen, China

^4^Graduate School, Guangxi University of Chinese Medicine, Nanning, China

^5^Department of General Medicine, Geriatric Hospital Affiliated with Wuhan University of Science and Technology, Wuhan, China

^6^Hunan Provincial Key Laboratory of the Research and Development of Novel Pharmaceutical Preparations, Changsha Medical University, Changsha, China

**^#^ Equal contributors**

***Corresponding author:**

**Liming Cao**

Department of Neurology,

The First Affiliated Hospital of Shenzhen University,

Shenzhen 518000, China

Phone No.: +8615017913715

Email: [caolm-2007@163.com](mailto:caolm-2007@163.com)

**ORCID:** https://orcid.org/0000-0003-2836-9347

**Online Resource Table 1 Summary of All Cases in the Study**

| Case/Time | Age/Sex | Chief complaint/main symptoms/positive signs | Medical history/Posture at onset | Main examination results | Treatment | Outcomes and/or follow-up |
| --- | --- | --- | --- | --- | --- | --- |
| Case 1/ Jan 12, 2024. | 40/M | Cough-associated syncope for 17 d.  The patient experienced syncope following severe coughing, with a frequency of up to 12 episodes daily. Syncope occurred during walking, driving, and seated rest and resulted in a forehead injury and a motor vehicle collision. Each syncopal episode lasted 3–4 s, without convulsions.  BMI = 26.12 kg/m^2^, with slightly coarse breath sounds bilaterally. | Allergic rhinitis, pulmonary infection, OSA, history of smoking /Standing or sitting position | WBC = 11.19×10⁹/L, adenovirus nucleic acid test (+), moderate sensitization to mixed dust mites. Blood gas analysis, liver/renal function, CRP, procalcitonin, serum IgE (–). CT imaging showed scattered mild inflammatory foci in the bilateral lungs. Polysomnography revealed severe OSA with predominant hypopnea events. Pulmonary function tests, bronchial provocation test, ECG, echocardiography, carotid ultrasonography, cranial MRI (–). Combined HUTT and TCD (+). | Clarithromycin, montelukast, and loratadine | Cough symptoms improved significantly, with one CS recurrence post-admission. No CS recurrence at the 8-mo follow-up. |
| Case 2/ Jan 22, 2024. | 39/M | Cough with expectoration for 2 wk, accompanied by 10 episodes of syncope. The patient experienced intermittent coughs plus a sore throat. Levofloxacin and cefaclor relieved the sore throat but aggravated the cough, which progressed to a severe dry cough. Syncope ensued immediately after severe coughing bouts; each episode lasted ~2 s, with a total of 10 CS episodes documented. Some episodes were accompanied by fist- clenching and tremors. BMI = 27.04 kg/m^2^, pharynx showed mild congestion. | Bronchial asthma, allergic rhinitis, hypertension, hyperlipidemia, pulmonary infection/Standing position | WBC = 13.22×10^9^/L, creatinine 153 umol/L, Mycoplasma pneumoniae IgG antibody (+). Severe sensitization to house dust mite/dust mite mix; Total serum IgE: 749.0 ng/mL. CT imaging showed a minor nodule in the left upper lobe. Carotid artery ultrasound showed carotid arteriosclerosis. ECG, echocardiography, cranial MRI, and EEG (–). HUTT (+). | Ceftriaxone, nebulized terbutaline, compound methoxyphenamine, montelukast, and lipid-lowering therapies. | The cough significantly improved.  A follow-up at 8 mo showed no CS recurrence. |
| Case 3/ Jan 22, 2024. | 43/M | Recurrent cough for 1 mo, accompanied by fever and syncope for 3 d. The patient initially had a paroxysmal cough with scant yellow, viscous sputum; the cough worsened 3 d later, accompanied by fever, and antipyretic and antitussive medications were administered. Two days later, he had a bout of continuous severe coughing, which induced syncope, with consciousness regained within seconds and no convulsions. BMI = 30.1 kg/m^2^. | Thyroid nodule, hepatic steatosis, OSA, pulmonary infection/Standing position | CRP = 9.97 mg/L; WBC, procalcitonin, liver function, blood glucose, arterial blood gas analysis, respiratory virus multiplex nucleic acid test, acid-fast bacilli, and fungal smear microscopy (–). CT imaging revealed right lower lobe inflammation and possible mild inflammatory foci in the left lung. Polysomnography showed OSA. ECG, echocardiography, carotid ultrasonography, cranial MRI, EEG (–). Combined HUTT and TCD (+). | Ceftriaxone, fluticasone, eucalyptol-limonene-pinene enteric capsules, and urate-lowering therapies. | The cough significantly improved. A follow-up at 8 mo showed no recurrence of CS. |
| Case 4/ Dec 14, 2024. | 61/M | Recurrent cough accompanied by syncope for 2 mo, with two syncopes. The patient initially experienced a sudden onset of severe coughing during a meal, followed by dyspnea after several coughs, culminating in syncope with a fall. Consciousness was regained within seconds, with no convulsions, urinary incontinence, or fecal incontinence. A recurrent cough with paroxysmal dyspnea persisted over the subsequent 2 mo, and syncope recurred 2 d before presentation during a meal-associated cough. BMI = 31.1 kg/m^2^. | Hypertension, coronary artery disease, hyperlipidemia, OSA, bronchial asthma, pulmonary infection/ Sitting position | Hemoglobin = 97 g/L. CT imaging showed scattered inflammatory changes in bilateral upper lobes and the right lower lobe and multiple small pulmonary nodules. MRI showed multiple ischemic foci and a cerebellomedullary cistern arachnoid cyst. Echocardiography showed a dilated ascending aorta, left atrial enlargement, ventricular septal thickening, and impaired left ventricular diastolic function. Carotid ultrasonography showed heterogeneous intima-media thickening of the bilateral carotid arteries. ECG showed complete right bundle branch block, left anterior fascicular block, and premature atrial contractions. HUTT (+). | Ceftriaxone, atorvastatin, telmisartan, clopidogrel, ketotifen, and levocetirizine. | The symptoms improved.  A follow-up at 6 mo showed no recurrence of CS. |
| Case 5/ Dec 30, 2023. | 72/F | Recurrent syncope for 1 yr, with over 30 episodes. Recurrent dizziness was elicited by head flexion or shaking. Syncope was induced on ≥ 10 occasions during defecation and approximately 10 times by severe coughing. Each syncopal event lasted several seconds, without convulsions or urinary incontinence. BMI = 24.14 kg/m^2^ | Hypertension, diabetes mellitus, hyperlipidemia,  Posterior circulation ischemia, sleep disorder, pulmonary nodule resection/Standing or sitting position. | CT imaging showed postoperative changes of pulmonary nodule resection. MRI showed mild white matter lesions. Cerebral DSA revealed mild stenosis of the right internal carotid artery C7 segment and left vertebral artery ostial stenosis. ECG showed atrial premature contractions. HUTT (–). | Rosuvastatin, enteric-coated aspirin, zolpidem, agomelatine, and tongtian oral liquid. | The dizziness improved.  A follow-up at 3 mo showed no recurrence of CS. |
| Ma Et al.  [9]  2011. | 38/M | Recurrent unconsciousness over the past 0.5 mo. Following exposure to low temperatures, the patient developed a cold accompanied by coughing. During the recent 0.5 mo, recurrent severe coughing episodes triggered unconsciousness, each lasting between 5 and 30 s. Upon regaining consciousness, the patient experienced pallor and dizziness, with a total of 15 such episodes. Bilateral coarse breath sounds were present on lung auscultation. | Upper respiratory tract infection/Unreported | Blood analysis and cranial MRI (–). Echocardiography revealed mild tricuspid regurgitation. | Oxygen, anti-infective, bronchodilation, and anti-asthmatic therapies. | Symptoms improved, and CS did not recur. |
| Wang Et al.  [10]  2015. | 39/M | Intermittent cough accompanied by syncope over a 2-wk period. Obesity. | Pulmonary infection, cerebral stroke, history of smoking/Unreported | Blood analysis, sputum culture, and 24-h ambulatory ECG (–). CXR showed a left lower lobe infection. CT imaging revealed a lacunar cerebral infarction. Pulmonary function tests showed a small airway ventilation impairment. | Codeine, expectorants and bronchodilators, and traditional Chinese herbal medicine therapies. | The cough had completely resolved at the 1-mo follow-up, with no recurrence of CS. |
|  | 22/F | CS lasting 3 d. The symptoms began 3 d following an argument with family members, manifesting as coughing followed by unconsciousness, accompanied by gurgling sounds in the throat, facial pallor, and diaphoresis. Consciousness was regained after approximately 1 min. Subsequently, exposure to irritating odors triggered coughing, which, in severe cases, leads to syncope. | Bronchial asthma/Unreported | ECG, cranial CT, and EEG (–). | Antitussive, expectorant, and traditional Chinese herbal medicine therapies. | Symptoms improved, and CS did not recur. |
| Bai Et al.  [11]  2007. | 62/M | Worsening chest tightness, shortness of breath, cough, and sputum production. On the night of admission, the patient experienced sudden syncope triggered by a severe coughing episode, with spontaneous recovery after approximately 2 min. However, he experienced multiple recurrences of CS during hospitalization. Cyanotic lips, barrel chest, diminished breath sounds in both lungs, and audible dry and moist rales were all present. | Silicosis, pulmonary infection/Unreported | WBC = 14.6×10⁹/L, neutrophil percentage = 78%. Sputum culture (+) for *Pseudomonas aeruginosa*. CXR showed stage I pneumoconiosis, pulmonary infection, and emphysema. | Amikacin, ceftazidime, aminophylline, ambroxol, and oxygen therapies. | Discharged after recovery. |
| Yu Et al.  [12]  2016. | 65/M | A 5-yr history of CS. The patient experienced weakness in the limbs and dimming of vision after paroxysmal severe coughing, progressing to unconsciousness lasting approximately 10–30 s, with a total of 12 episodes recorded. Coarse breath sounds in both lungs. | Chronic bronchitis/Unreported | Blood analysis, CXR, ECG, EEG, cranial CT (–). TCD showed a mild reduction in cerebral blood flow velocity. | Corticosteroids, antibiotics, and antitussive therapies. | During the 5-yr follow-up, the longest interval without syncope was 7 mo, and the mean interval between syncopal episodes was approximately 5 mo. |
|  | 60/M | Cough-associated syncope of 3-d duration. Following exposure to low temperatures, the patient developed a headache and cough. Unconsciousness followed several consecutive coughing episodes, lasting approximately 40 s–1 min before recovery. Syncope was also triggered at times by mild coughing, with a frequency of 7–8 episodes per day. Obesity, with scant dry rales audible in both lungs. | Chronic cough and wheezing, acute bronchitis/Unreported | Neutrophil percentage = 80%. CXR showed acute bronchitis. EEG, ECG, echocardiography, cranial CT (–). | Ceftriaxone, levofloxacin, and antitussive therapies. | Discharged after recovery. |
| Xu Et al.  [13]  2007. | 46/M | Frequent episodes of irritative dry cough, with syncope occurring during severe coughing paroxysms for 2 mo. Five syncopal episodes occurred before hospitalization, with two additional episodes occurring during the 10-d hospital stay. Syncope typically followed several consecutive coughs, with consciousness regained after approximately 10–20 s, followed by fatigue. | Undifferentiated lung carcinoma, pulmonary infection/Unreported | Blood glucose, ECG, and EEG (–). | Antitussives, expectorants, bronchodilators, and anti-infective therapy. Systemic chemotherapy with paclitaxel and cisplatin. | Symptoms improved, and CS did not recur. |
|  | 73/M | Two episodes of CS within 8 d. One episode occurred during nocturnal coughing, and the other during coughing while using the bathroom. Before coughing started, the patient reported chest tightness and shortness of breath. Following 5–6 consecutive coughs, he experienced dimming of vision followed by syncope, each episode lasting approximately 10–25 s. Upon regaining consciousness, he reported dizziness and fatigue, with complete resolution of symptoms after approximately 10 min. | Lung adenocarcinoma, pulmonary infection/Unreported | Blood glucose, ECG, and EEG (–) | Antitussives, expectorants, bronchodilators, and anti-infective therapy. Systemic chemotherapy with paclitaxel and cisplatin. | Discharged after recovery. |
| Teng Et al.  [14]  2017. | 39/M | Recurrent cough and expectoration for 10 yr, which had worsened over the 3 mo before admission. Symptoms exacerbated with physical exertion or cold exposure, and occasional hemoptysis was reported. After admission, the patient was diagnosed with pulmonary aspergillosis. Multiple syncopal episodes occurred during coughing, each triggered by paroxysmal severe coughing, with consciousness regained after approximately 1–2 min. | Pulmonary aspergillosis/Unreported | CT imaging revealed a significant increase in the suspected aspergilloma within the cavity of pulmonary aspergillosis in the right lung. Multiple osteolytic changes in the right ribs were more pronounced, accompanied by volume loss of the right lung. | Itraconazole, piperacillin sodium and tazobactam sodium, corticosteroids, asmeton, and myrtol. | Cough and expectoration improved after 2 wk of treatment. |
| Hao Et al.  [15]  2015. | 40/M | Recurrent cough accompanied by syncope for over 10 d. The cough developed following cold exposure and was associated with scant sputum. Treatment for 1-wk with anti-inflammatory, antitussive, and expectorant medications yielded minimal improvement, after which CS episodes began. Each syncopal episode lasted approximately 2–10 s.  BMI = 22.5 kg/m², with slightly coarse breath sounds in both lungs. | Allergic rhinitis, cough-variant asthma/Unreported | ECG, echocardiography, cervical vascular ultrasonography, gastroscopy, paranasal sinus CT, EEG, TCD, cranial MRI (–). CXR showed increased bilateral pulmonary markings. Pulmonary function tests revealed a predicted FEV1 ≥70%; bronchial provocation test indicated airway hyperresponsiveness with mild airway inflammation. | Azithromycin, ambroxol, loratadine, theophylline, Ventolin, budesonide/formoterol, montelukast, and licorice tablets. | No recurrent syncope at the 1-wk follow-up. |
| Qiu Et al.  [16]  2018. | 56/M | Paroxysmal syncope for 9 yr, aggravated over the 7-wk period before admission. After cold exposure, the patient had episodic coughing that once caused breath-holding and subsequent syncope. Over the following 2 yr, CS recurred consistently after cold exposure, at an average frequency of once every 3–5 d. Symptoms worsened 3 wk pre-admission, with daily syncope; two syncopal episodes occurred during hospitalization, both accompanied by limb myoclonus and tachycardia. Coarse breath sounds in both lungs. | Cough-variant asthma, hypertension, coronary heart disease, diabetes mellitus, cerebral stroke, history of smoking/Unreported | Serum IgE elevated at 5330 ng/mL. WBC (–). CT imaging revealed multiple lacunar infarcts. Cranial CTA showed small calcified plaques in bilateral internal carotid arteries and mild calcific changes in cerebral arteries. Carotid ultrasonography indicated atherosclerotic plaques in bilateral carotid arteries. EEG, ECG, echocardiography, 24-h ambulatory ECG, ambulatory blood pressure monitoring, HUTT (–). Bronchial dilation test (+). | Compound methoxyphenamine capsules, salmeterol, and fluticasone powder inhaler. | One episode of CS recurrence at the 2-mo follow-up. |
| Liu Et al.  [17]  2016. | 60/M | Intermittent cough and expectoration for over a 2-mo period, worsened with occasional syncope over the wk before admission. After cold exposure, the patient developed cough, expectoration, wheezing, and chest tightness with progressive worsening; sudden syncope occurred during a coughing episode, with unconsciousness lasting ~1 min. He subsequently had multiple severe cough-triggered syncopal episodes, with a total of 6 CS events pre-admission. Coarse breath sounds in both lungs, along with scant moist rales audible at both lung bases. | Chronic bronchitis, coronary heart disease, chronic lymphocytic thyroiditis/Unreported | CRP elevated at 14.62 mg/L. WBC (–). CT imaging showed chronic bronchitis with emphysema. TCD showed decreased blood flow velocity in the bilateral middle cerebral and basilar arteries. Cranial MRI showed mild demyelinating changes in the bilateral centrum semiovale. A 24-h ambulatory ECG revealed sinus tachycardia; 24-h ambulatory blood pressure monitoring (–). Echocardiography showed aortic sclerosis, ascending aortic dilation, and pulmonary hypertension. | Moxifloxacin, compound methoxyphenamine, asarone and codeine, nimodipine, and aspirin. | No further CS occurred at the 2-mo follow-up. |
| Liu Et al.  [18]  2012. | 32/M | Episodic unconsciousness over a 3-yr period. The patient experienced recurrence of CS, each lasting approximately 10 to 20 s. A week before admission, after alcohol consumption, he again developed a persistent choking cough followed by sudden unconsciousness lasting approximately 15 s. Coarse breath sounds in both lungs with audible dry and moist rales. | Bronchial asthma, history of smoking/Unreported | Blood analysis, ECG, EEG, echocardiography, HUTT (–). Abdominal ultrasonography revealed hepatic steatosis. CT imaging showed increased bilateral lung markings. MRI demonstrated bilateral periventricular and corona radiata white matter demyelination. Magnetic resonance angiography showed basilar artery tortuosity. | Bronchodilation and anti-asthmatic treatment. | No recurrent syncope during the follow-up. |
| Que Et al.  [19]  2006. | 60/M | CS of 2-d duration. The patient developed a headache and cough after cold exposure. Treatment with roxithromycin was ineffective, and the cough worsened. After several consecutive coughs, sudden unconsciousness occurred, lasting approximately 30 s–1 min before spontaneous recovery. No limb convulsions or incontinence occurred during the episode. Obesity, with scant dry and moist rales audible in both lungs. | Acute bronchitis/Unreported | Neutrophil percentage = 80%. CXR showed acute bronchitis. Abdominal ultrasonography revealed mild fatty liver. EEG, ECG, echocardiography, cranial CT (–). | Cefotaxime, levofloxacin, bromhexine, and codeine | No CS occurred at the 4-yr follow-up. |
| Zhang Et al.  [20]  2009. | 46/M | Intermittent severe cough accompanied by syncope for over 20 d. Since the onset, the patient had experienced cough, with scant white sputum. Paroxysmal severe coughing was accompanied by a transient sensation of mental blankness, haziness, and a feeling of bodily levitation, followed by unconsciousness lasting 10–20 min before spontaneous recovery. | Bronchitis, history of smoking/Unreported | Blood analysis, sputum culture, cranial CT, EEG (–). CXR showed bronchitis. TCD showed a mild reduction in cerebral blood flow. Bronchoscopy revealed bronchitis-consistent changes. | Bromhexine, saxifrage extract, theophylline sustained-release tablets, and ampicillin yielded a suboptimal response. Subsequently, anti-infective was combined with vitamin K supplementation. | No recurrent syncope during the follow-up. |
| Zhao Et al.  [21]  2021. | 65/M | Paroxysmal CS for over a 3-yr period, aggravated over the week after admission. Syncope consistently followed coughing, each lasting several seconds with limb tremors, dyspnea, chest tightness, heartburn, and precordial discomfort. The patient had frequent baseline dizziness and headaches. Pre-admission CS episodes occurred 7–8 times per day; recurrent CS 1 wk pre-admission prompted hospitalization.  Coarse breath sounds in both lungs, with wheezing audible. | Pulmonary infection, hypertension, coronary heart disease, cerebral stroke, diabetes mellitus, history of smoking/Unreported | CRP = 10.28 mg/L, serum IgE elevated at 304.0 IU/mL. WBC (–). ECG showed abnormal Q waves in inferior leads and a Qr pattern in V_1_. Echocardiography demonstrated ventricular septal hypertrophy and impaired left ventricular diastolic function. CT imaging showed bronchitis, localized emphysema, and scattered chronic inflammatory changes in bilateral lungs. Pulmonary function tests revealed moderate restrictive ventilatory dysfunction. | Ceftriaxone sodium, doxofylline, compound methoxyphenamine, | Symptoms improved, and CS did not recur. |
| Li Et al.  [22]  1995. | 67/M | Recurrent CS over a 3-mo period. Following low-temperature exposure, he developed a cough productive of white, viscous sputum. Symptoms worsened 1 mo beforeadmission, accompanied by paroxysmal severe coughing with thick sputum. After such coughing paroxysms, the patient experienced generalized numbness, weakness, and sudden unconsciousness lasting approximately 30 sec. A total of three such episodes occurred. Scattered dry rales in both lungs. | Acute bronchitis, pulmonary infection/Unreported | Blood analysis, CT, EEG, ECG (–). CXR showed increased and thickened markings in the bilateral lower lungs with 2–3 small patchy opacities, suggestive of bronchitis with secondary infection. TCD revealed reduced blood flow in the right vertebral artery system. | Cephalosporin and ampicillin. Antitussive/expectorant medication and nebulized therapies. | No recurrence of CS during the 7-mo follow-up. |
| Zhao Et al.  [23]  1999. | 54/M | Worsening cough and expectoration over a 20-d period, accompanied by transient unconsciousness over the 7 d before admission. The cough was paroxysmal and spasmodic, with scant white viscous sputum. During episodes of consecutive coughing, facial flushing occurred, followed by unconsciousness lasting several seconds before spontaneous recovery. BMI = 32 kg/m² and diminished breath sounds in both lungs. | Bronchitis/Unreported | CXR indicated bronchitis in both lungs. Cranial and thoracic CT, ECG, EEG, TCD, and ambulatory ECG (–). | Cefuroxime | CS did not recur after 1 wk of treatment. |
| Li Et al.  [24]  2020. | 63/M | Intermittent cough and expectoration for 2 wk, aggravated with syncope over the 3 d beforeadmission. Post-low temperature exposure, the patient had an intermittent productive cough with yellow viscous sputum, plus dizziness, headache, fatigue, and low-grade fever. 3-d pre-admission, the cough worsened with scanty white frothy sputum that was hard to expectorate; three consecutive coughing bouts induced unconsciousness with mild limb twitching, with spontaneous recovery after ~5–8 s.  BMI = 24 kg/m², with coarse breath sounds in both lungs. | Upper respiratory tract infection, hypertension, diabetes mellitus, history of smoking/Unreported | WBC = 10.5×10⁹/L, sputum culture, 24-h ambulatory ECG, HUTT, bronchial dilation test (–). TCD indicated cerebral arteriosclerosis. Ambulatory EEG showed mild abnormalities. Echocardiography revealed left atrial enlargement, mild mitral regurgitation, and mild tricuspid regurgitation. CT imaging showed interstitial changes in the left lower lingular segment and bilateral lower lobes. Pulmonary function tests indicated moderate restrictive ventilatory dysfunction. | Antitussive, anti-infective, expectorant, and bronchodilation therapies. | Discharged after recovery. |
| Li Et al.  [25]  2010. | 65/M | Recurrent cough for 1 wk accompanied by two syncopal episodes. The patient developed an upper respiratory infection with paroxysmal cough and fever before admission. 2 d before administration, two episodes of CS occurred, each lasting approximately 10–30 s. After 2 d of hospitalization, another syncopal episode was triggered by a sudden paroxysmal cough, lasting approximately 15 s before spontaneous recovery. Pharyngeal congestion and grade 2 tonsillar enlargement. | Acute pharyngitis/tonsillitis/Unreported | WBC = 10.5×10⁹/L, neutrophil percentage = 80%. CXR, ECG, echocardiography, cranial CT (–). TCD showed mildly decreased cerebral blood flow velocity. | Penicillin, nebulized therapy, and antitussive/expectorant medication. | No recurrence of CS during the 1-mo follow-up. |
| Wang Et al.  [26]  2023. | 59/M | Paroxysmal syncope over a 2-d period. The patient experienced an episode of CS before admission, with recovery after approximately 10 s and no limb convulsions. Multiple previous syncopes had occurred during severe coughing, one of which resulted in a fall causing head injury. BMI = 24.6 kg/m², soft tissue swelling with subcutaneous hemorrhage around the left eye, coarse breath sounds in both lungs, and audible moist rales. | Upper respiratory tract infection, coronary heart disease, OSA, hypertension, diabetes mellitus, cerebral stroke/Unreported | *Mycoplasma pneumoniae* test result was weakly positive. CRP = 12.10 mg/L. Echocardiography showed impaired left ventricular diastolic function. CT imaging revealed patchy increased opacities in the left lower lobe. Cranial CT imaging demonstrated a subcutaneous hematoma in the left frontal region. ECG, TCD, and CTA of the pulmonary arteries (–). *Mycoplasma pneumoniae* tested weakly (+). Echocardiography showed impaired left ventricular diastolic function. CT imaging revealed patchy increased opacities in the left lower lobe and a left frontal subcutaneous hematoma. | Budesonide, acetylcysteine, montelukast sodium, dextromethorphan, and guaifenesin syrup. Moxifloxacin, methylprednisolone, antiplatelet, lipid-lowering, antihypertensive, and  hypoglycemic therapies. | No recurrence of CS during the 1-mo follow-up. |
| Xu Et al.  [27]  2014. | 92/M | Recurrent cough and expectoration for over 20 yr, aggravated with wheezing over the past 2 d. Post-low-temperature exposure, the patient had a productive cough with pale yellow viscous sputum, severe nocturnal wheezing, and dyspnea precluding supine position. Three CS episodes occurred (each ~10–30 s), with multiple further episodes documented during hospitalization.  BMI = 30.47 kg/m², coarse breath sounds in both lungs, scattered moist rales in the lower lungs bilaterally, and wheezing audible over the right lung. | Chronic bronchitis, pulmonary infection, diabetes mellitus, hypertension, coronary heart disease, Alzheimer’s disease/Sitting position. | WBC = 10.8 × 10⁹/L, blood glucose level = 11.0 mmol/L. CT imaging showed inflammation in the right lower lobe. MRI demonstrated multiple cerebral infarcts and cerebral atrophy. ECG indicated complete right bundle branch block. | Meropenem, moxifloxacin,  fluconazole, ambroxol, aminophylline | No recurrence of CS during the 4-mo follow-up. |
| Gao Et al.  [28]  2008. | 78/M | Recurrent cough and wheezing for 40 yr, exacerbated with CS over the 20 d before admission. The cough and wheezing worsened after cold exposure, with sputum that was difficult to expectorate. During episodes of severe coughing, transient unconsciousness accompanied by limb convulsions occurred, with spontaneous recovery after approximately 1–2 min. Diminished breath sounds in both lungs, with moist rales audible at the lung bases. | Chronic bronchitis, pulmonary infection/Unreported | Neutrophil percentage = 79%. CXR showed increased bilateral lung markings with small patchy shadows in the lower lungs. Cranial CT (–). Ambulatory ECG revealed occasional ventricular premature contractions and myocardial ischemia. Pulmonary function tests indicated obstructive ventilatory dysfunction. | Anti-infective and bronchodilation/anti-asthmatic treatment. | No recurrence of CS during the 3-mo follow-up. |
|  | 49/M | Intermittent cough and wheezing for 20 yr, exacerbated with CS over the 3 d before admission. Three days before admission, after cold exposure, the cough and wheezing worsened and were accompanied by paroxysmal syncope, transient unconsciousness, and limb convulsions. A wheezing appearance, barrel chest, widespread wheezing audible throughout both lungs, and moist rales in the left lower lung field. | Persistent bronchial asthma, community-acquired pneumonia/Unreported | WBC = 20.0×10⁹/L, neutrophil percentage = 89%. Sputum culture (+) for *Escherichia coli*. CXR showed accentuated lung markings and patchy opacities in the left lower lobe. Pulmonary function tests indicated mild obstructive ventilatory dysfunction combined with mild restrictive ventilatory dysfunction. | Anti-infective and bronchodilation/anti-asthmatic treatment. | No recurrence of CS during the 2-mo follow-up. |
|  | 53/M | Intermittent cough accompanied by CS over the 0.5 mo before admission. after cold exposure, the patient developed a cough along with paroxysmal syncope, transient unconsciousness, and limb convulsions. Each syncopal episode lasted several seconds before spontaneous recovery. | Upper respiratory tract infection/Unreported | Blood analysis (–). Pulmonary function tests indicated mild obstructive ventilatory dysfunction. Cervical vascular ultrasonography revealed an atherosclerotic plaque in the right carotid artery with local luminal narrowing. | Antitussive, expectorant vasodilator, and lipid-lowering therapies. | No recurrence of CS during the 2-mo follow-up. |
| Huang et al.  [29]  2002. | 50/M | Numbness in both hands and dimming of vision following paroxysmal severe coughing, which progressed to unconsciousness. Spontaneous recovery occurred within tens of seconds, followed by palpitations, diaphoresis, and generalized weakness. One episode resulted in accidental head injury. Over a 5-yr disease course, a total of 12 episodes occurred, predominantly during winter. Coarse breath sounds in both lungs. | Bronchial asthma, chronic bronchitis/Unreported | CXR, ECG, EEG, and cranial CT (–). | Corticosteroids, anti-infective, and antitussive medication. | Discharged after recovery. |
|  | 61/M | Syncope triggered by severe coughing secondary to bronchitis for 1 yr. Six episodes occurred over this period. Each event was characterized by unconsciousness following intense coughing, resulting in falls and facial pallor, with a duration of 1–3 min. Upon recovery, the patient experienced profuse generalized diaphoresis and palpitations. Baseline blood pressure was normal but elevated significantly after severe coughing, reaching a maximum recorded value of 170/112 mmHg. | Bronchitis/Unreported | CXR showed coarse lung markings. ECG, EEG, cranial CT, and cervical vascular color ultrasonography (–). | Anti-infective and antitussive medication. | Discharged after recovery. |
|  | 59/M | An 8-yr history of CS, totaling 11 episodes. During attacks, he experienced dimming of vision followed by falls and unconsciousness. Postictal symptoms included generalized diaphoresis. Elevated blood pressure (140–160/90–96 mmHg) following two of the episodes. | - | EEG, ECG, cervical vascular ultrasonography, and cranial CT (–). | Anti-infective and antitussive medication | No recurrence of CS during the 2-yr follow-up. |
| Fang Et al.  [30]  2025. | 54/M | Cough accompanied by transient unconsciousness for 5 d. Following low-temperature exposure, the patient developed a paroxysmal cough productive of yellow mucoid sputum. During severe coughing episodes, significant breathlessness and facial flushing occurred, along with transient impairment of consciousness manifested by upward deviation of the eyes, limb convulsions, and frothing at the mouth. These episodes lasted several seconds before spontaneous resolution and were followed by a mild headache. Multiple episodes occurred daily.  BMI = 34 kg/m², and coarse breath sounds in both lungs. | Pulmonary infection, diabetes mellitus, OSA/Unreported. | Blood analysis, sputum culture, ambulatory ECG, ambulatory blood pressure monitoring, 24-h ambulatory EEG, craniocervical CT, echocardiography, and HUTT (–). Carotid ultrasonography indicated bilateral carotid intima-media heterogeneous thickening and mild right vertebral artery stenosis. CT imaging showed multiple inflammatory foci in bilateral lungs. MRI demonstrated scattered punctate ischemic foci in bilateral frontal lobes and cerebral atrophy. Nasopharyngolaryngoscopy revealed right vocal cord inflammation and chronic pharyngitis. Sleep monitoring indicated mild OSA, predominant hypopnea events, and moderate nocturnal hypoxemia. | Ceftriaxone, cefoperazone-sulbactam, ceftizoxime, peramivir, doxycycline, montelukast, methylprednisolone, budesonide, terbutaline, ipratropium bromide, oxcarbazepine, and codeine. | No recurrence of CS during the 6-mo follow-up. |
| Chen Et al.  [31]  2010. | 84/M | Recurrent CS for over 1 yr, with exacerbation over the month before admission. The patient initially experienced unconsciousness following mild coughing, lasting several tens of seconds before spontaneous resolution. 1-mo before admission, syncope recurred, with each episode preceded by severe coughing. Three CS episodes occurred 0.5 d before admission, prompting hospitalization. Diminished breath sounds in both lungs. | COPD, hypertension, history of smoking/Unreported | Blood analysis, EEG, video EEG, echocardiography, ambulatory ECG (–). MRI demonstrated ischemic foci in the bilateral frontal and parietal lobes with cerebral atrophy. Craniocervical arterial color ultrasonography indicated carotid arteriosclerosis. CT imaging revealed bilateral lung changes consistent with chronic bronchitis and emphysema, scattered linear opacities in the lungs, and left lower pleural thickening. | Anti-infective and bronchodilation/anti-asthmatic treatment. | Condition improved, and subsequent intermittent follow-ups showed no recurrence. |
| Pang Et al.  [32]  2013. | 62/M | Sudden onset of unsteadiness and three falls in 1 d. During activity, the patient lost control of posture and fell to the ground three times, each episode accompanied by brief unconsciousness and abrasions at the impact sites. On the third day of hospitalization, a similar fall occurred during morning washing, preceded by severe coughing in each instance.  Coarse breath sounds in both lungs, with no audible dry or moist rales. | Hypertension, cerebral stroke/standing position. | Blood analysis, ECG, EEG, cervical vascular ultrasonography (–). CXR showed slightly increased lung markings. TCD indicated mildly decreased cerebral blood flow. CT imaging revealed an old cerebral infarction in the left basal ganglia. MRI demonstrated multiple ischemic lacunar foci in the bilateral corona radiata. | Antitussive, expectorant, bronchodilation, nebulized corticosteroid, antiplatelet, anticoagulant, and antihypertensive therapies. | No recurrence of CS during the 1-mo follow-up. |
| Wang Et al.  [33]  1993. | 50/M | The patient had experienced one episode of CS 2 yr earlier, with spontaneous recovery and no other associated symptoms. During the medical evaluation at that time, asymmetric upper limb blood pressure, leading to a diagnosis of "subclavian steal syndrome." CS recurred 6 mo earlier, initially at a frequency of approximately once per week, with progressive worsening that has now impaired his ability to perform normal work. | Subclavian artery steal syndrome, history of smoking/Unreported | Pulmonary function test (–). Cervical vascular ultrasonography showed complete occlusion of the left common carotid artery, right internal carotid and right vertebral arteries, severe stenosis of the right subclavian artery, and left vertebral artery hypoplasia. CXR revealed left ventricular enlargement. ECG indicated left ventricular hypertrophy; 24-h ambulatory ECG demonstrated ventricular premature contractions. CT imaging showed mild hypodensities in the bilateral cerebral white matter. | Revascularization of the left common carotid artery and left subclavian artery. | No recurrence of CS during the 14-mo follow-up, and the patient only presented with mild cough accompanied by slight dizziness. |
| Li Et al.  [34]  2018. | 83/M | Paroxysmal CS over a 0.5-mo duration. The patient developed cough and nasal congestion accompanied by syncope 0.5 mo before admission after cold exposure, resulting in a fall to the ground with associated pallor and diaphoresis. Consciousness was regained after approximately 10 s, with abrasions noted at the impact sites. Subsequently, cough and expectoration worsened, and syncope recurred frequently, totaling four episodes, none of which involved convulsions.  Ecchymoses on the left forehead, periorbital area, and limbs. Coarse breath sounds in both lungs, along with scant moist rales. | Hypertension, COPD, pulmonary infection, chronic bronchitis/Unreported | ECG, ambulatory ECG, carotid ultrasonography (–). Echocardiography demonstrated aortic valve calcification with mild insufficiency. CT imaging revealed a left frontal subcutaneous hematoma, increased density in the posterior falx cerebri, and cerebral atrophy. Contrast-enhanced CT imaging showed bilateral diffuse emphysema and left-sided pleural effusion. | Antihypertensive, lipid-lowering, anti-infective, anti-asthmatic, bronchodilation and expectorant therapies. | Discharged after recovery. |
| Zhong Et al.  [35]  2000. | 30/M | Recurrent cough over a 1-mo period accompanied by CS. During the month before admission, syncope occurred 1–2 times daily, all triggered by severe coughing. Manifestations included facial congestion, sudden unconsciousness, fixed gaze, and falls. Recovery occurred within 3–5 s.  Weight = 95 kg, scant dry and moist rales audible in both lungs, conjunctival congestion in both eyes | Left lower lobe pneumonia/Unreported | WBC = 14.2×10⁹/L, neutrophil percentage = 80%. CXR showed coarse lung markings; CT imaging indicated left lower lobe inflammation. EEG showed mild abnormalities, and epilepsy was ruled out. Cranial CT, ECG, echocardiography, ambulatory ECG (–). | Anti-infective,  Antitussive,  bronchodilation,  anti-allergic,  and traditional Chinese medicine therapies. | The patient recovered fully after a 51-d hospitalization. |
| Luo Et al.  [36]  2019. | 55/M | Cough and expectoration for 1 wk, accompanied by multiple episodes of CS. The patient developed a cough with copious, thin, white sputum after cold exposure. During severe coughing, syncope occurred, lasting for several seconds, with multiple episodes daily, preceded by mild dizziness.  Slightly coarse breath sounds in both lungs. | Community-acquired pneumonia, hypertension, OSA, coronary heart disease, cerebral stroke, history of smoking /Unreported. | WBC = 14.47×10⁹/L. CT imaging showed inflammation in the right middle lobe, anterior basal segment of the right lower lobe, and lingular segment of the left upper lobe. Sleep monitoring indicated severe OSA. ECG (–). Echocardiography demonstrated segmental left ventricular wall motion abnormalities, mild aortic and tricuspid valve insufficiency, and mild pulmonary hypertension. CT imaging showed multiple lacunar infarcts in the bilateral basal ganglia. TCD indicated reduced blood flow velocity in the intracranial segment of the left vertebral artery and the basilar artery. | Ceftizoxime, ambroxol, dextromethorphan, hydrobromide, agents to improve circulation, lower blood pressure, and reduce lipids. | No recurrence of CS during the 6-mo follow-up. |
| Zhu Et al.  [37]  2014. | 86/M | Cough and expectoration for 2 wk accompanied by chest tightness. Cough and scant yellow viscous sputum. Following coughing, chest tightness and shortness of breath occurred. During a coughing episode, sudden syncope developed, lasting several seconds before recovery.  Coarse breath sounds in both lungs, with scant moist rales audible in the right lung. | Pulmonary infection, rheumatic heart disease, atrial fibrillation/Unreported | Echocardiography revealed rheumatic heart disease with left atrial enlargement, mitral stenosis complicated by mild insufficiency, aortic valve insufficiency, trace tricuspid regurgitation, and a pulmonary artery systolic pressure of 39.5 mmHg. ECG demonstrated atrial fibrillation with T-wave changes. | Ceftazidime, moxifloxacin, ambroxol, gelomyrtol, budesonide, ipratropium bromide, diprophylline, dexamethasone, bumetanide, and spironolactone | The frequency of CS episodes decreased. |
| Li Et al.  [38]  2014. | 65/M | Recurrent cough for 3 yr, accompanied by paroxysmal CS over the year before admission. The patient had experienced paroxysmal cough for 3 yr, with fluctuating severity. Over the year before admission, the cough has worsened, particularly during meals, often followed by several bouts of severe coughing leading to sudden syncope lasting tens of s before recovery. More than 40 such syncopal episodes occurred.  A short, stocky body habitus, pharyngeal congestion. | Cough-variant asthma, GERD, chronic bronchitis/Sitting position. | Fiberoptic bronchoscopy, chest CT, cranial MRI (–). Gastroscopy revealed chronic superficial gastritis; histopathological examination confirmed moderate chronic superficial gastritis with negative *Helicobacter pylori*. 24-h esophageal pH monitoring demonstrated mild-to-moderate pathological acid reflux in the lower esophagus. | Initial anti-infective and antitussive therapy failed to relieve symptoms; esomeprazole magnesium and mosapride were added. | No recurrence of CS during the 1-yr follow-up. |
| Fan Et al.  [39]  2011. | 27/M | Intermittent CS over a 3-d period. Following low-temperature exposure, the patient developed a sore throat and cough with scant sputum. Severe coughing led to syncope with unconsciousness, lasting 1–2 min before resolution. Five to six episodes occurred over the 3-d before admission, resulting in falls and facial injuries. The occurrence of CS predominantly during nocturnal sleep raises suspicion for possible gastroesophageal reflux following sleep.  An obese body habitus, left supraorbital swelling, nasal ecchymosis, pharyngeal congestion, and coarse breath sounds in both lungs. | Acute bronchitis, GERD/Unreported | WBC = 10.62×10⁹/L. CXR showed increased and hazy lung markings, suggestive of bronchitis. Cranial CT (–). 24-h esophageal pH monitoring (+). | Despite 1-wk treatment with cefoperazone-sulbactam and doxofylline, nocturnal episodes of CS persisted during sleep; domperidone and omeprazole were then added. | No recurrent syncope during the follow-up. |
| Ci Et al.  [40]  2024. | 67/M | Recurrent CS for over 20 d. The patient had a pulmonary infection before admission and a positive right Babinski sign. | Pulmonary infection, cerebral stroke, hypertension, occlusion of the internal carotid and vertebral arteries, history of smoking/Unreported | Blood analysis (–). Craniocervical CTA showed near-total occlusion of both internal carotid arteries and occlusion of the V1–V2 segments of both vertebral arteries. MRI revealed old cerebral infarctions in the left centrum semiovale and corona radiata. CT perfusion imaging indicated delayed perfusion in the bilateral frontal and parietal lobes. Cranial DSA demonstrated complete occlusion at the origins of both internal carotid arteries and both vertebral arteries. | Anti-infective and bronchodilation/anti-asthmatic therapies.  Dual antiplatelet therapy with aspirin and clopidogrel.  Lipid-lowering therapy with simvastatin. | No recurrence of CS during the 12-mo follow-up. |
| Hu Et al.  [41]  2017. | 62/M | Recurrent paroxysmal dyspnea for over 20 yr, exacerbated with syncope for 1 d. For >20 y, the patient had recurrent paroxysmal dyspnea with low-grade fever no hemoptysis or night sweats. One day before admission, sudden dyspnea with CS developed on morning waking, lasting ~5 min with spontaneous resolution and no limb convulsions. Mild cyanosis on the lips and nail beds. Barrel chest, hyperresonance on percussion of both lungs, coarse breath sounds with audible dry and moist rales on auscultation, significant enlargement of the right cardiac dullness downward and laterally, and clubbing of the fingers and toes. | Kartagener syndrome, bronchial asthma/Unreported | Chest and abdominal CT imaging revealed dextrocardia, complete situs inversus of thoracic and abdominal organs, slightly increased bilateral lung markings, and bronchiectasis. Cranial CT, ECG, echocardiography (–). | Antibiotics, bronchodilators, and corticosteroids. | Discharged after recovery. |
| Xu Et al.  [42]  2015. | 43/M | Acid reflux for 2 mo and intermittent syncope for 1 wk. During the illness, the patient experienced recurrent acid reflux and retrosternal burning pain, occasional dry cough, and over the past wk, intermittent syncope accompanied by falls. Some episodes presented only as dimming of vision, lasting 5–20 s before spontaneous resolution, with a frequency of 2–4 episodes daily. Each episode was preceded by coughing, characterized as choking and severe, and each coughing spell was consistently preceded by noticeable acid reflux.  An obese body habitus. | GERD/Unreported | Blood analysis, chest CT, cranial CT, ECG, EEG, TCD (–). Gastroscopy revealed reflux esophagitis (Grade B) and chronic superficial gastritis with erosion. | Omeprazole and mosapride | No recurrence of CS during the 2-mo follow-up. |
| Liang Et al.  [43]  2020. | 44/M | Recurrent syncope over a 1-wk period. Following mild coughing triggered by low temperature exposure, he experienced syncope, with recovery after 1–2 min. These episodes occurred 5–10 times per day, all induced by coughing. During hospitalization, frequent CS episodes were observed, predominantly triggered by consecutive coughing, at a frequency of 3–4 times per day, occurring in both upright and supine positions.  BMI = 27.50 kg/m², grade I tonsillar enlargement, and pharyngeal congestion | Chronic pharyngitis, pulmonary infection, OSA, history of smoking/Standing or supine position. | Blood analysis, sputum culture, cranial MRI, magnetic resonance angiography, long-term video EEG (–). CT imaging showed bilateral pulmonary fibrotic foci. ECG and ambulatory ECG revealed sinus tachycardia. Echocardiography demonstrated mild tricuspid regurgitation and impaired left ventricular relaxation. Sleep monitoring indicated severe OSA, predominant obstructive events, accompanied by severe hypoxemia. Nasopharyngolaryngoscopy revealed pharyngeal narrowing, snoring signs, and chronic pharyngolaryngitis. HUTT (+). | Acyclovir, cefmetazole, cefoperazone-tazobactam, aminophylline, paracetamol dihydrocodeine, gabapentin, metoprolol, midodrine, and oryzanol | At the 1-mo follow-up,the patient reported no syncope during the first 2 wk of midodrine treatment. Syncope recurred after drug withdrawal but with a significantly lower frequency than at pre-treatment. |
| He Et al.  [44]  2014. | 45/M | Cough for 1 yr and small cell lung cancer of the left lower lobe for 10 mo. Over the 2 mo before admission, the cough became frequent, with white frothy sputum that was difficult to expectorate. Prior to admission, the patient had experienced two episodes of CS, each lasting several seconds, and one fall resulting in minor head injury. During hospitalization, three additional CS episodes occurred. | Small-cell lung cancer, pulmonary infection/Unreported | Blood analysis (–). CXR indicated inflammation in the left lower lobe. Cranial MRI, bone scintigraphy, and abdominal ultrasonography showed no evidence of tumor metastasis. | Piperacillin-sulbactam, codeine, ambroxol, budesonide, terbutaline, chemotherapy with etoposide and oxaliplatin. | Discharged after recovery. |
| Zhang Et al.  [45]  2017. | 61/M | Recurrent syncope for 10 yr, exacerbated over the past 0.5 mo. 10 yr before admission, the patient experienced choking while eating, which caused breath-holding, subsequent unconsciousness, and a fall with facial flushing, limb convulsions, and cold sweats, with consciousness recovery in ~30 s to 1 min. Over the 0.5 mo before admission, recurrent syncope developed, each triggered by 5–6 bouts of coughing and breath-holding, accompanied by limb convulsions, spontaneous consciousness recovery in 1–2 min, and a frequency of 2–8 episodes per day. | Anxiety state, sleep disorders/Unreported | Chest radiographs showed coarse lung markings. | Eszopiclone and codeine | Discharged after recovery. |
| Mangubat Et al.  [46]  2014. | 54/M | Progressive orthopnea and multiple episodes of CS. Two traffic accidents had occurred as a result of CS. Over the 1.5 yr before admission, the patient developed progressive gait instability, subjective quadriparesis, dysphagia, headache, and hoarseness. Bilateral exotropia with nystagmus, downbeat nystagmus on downward gaze, hoarseness, and weakness of palatal elevation. Diffuse hyperreflexia was present, accompanied by ataxia and impaired gait. | Chiari malformation type I, atlanto-occipital assimilation, OSA/Unreported | Cervical MRI demonstrated Chiari malformation type I, associated with atlanto-occipital assimilation resulting in a narrowed foramen magnum. | Chiari decompression, duraplasty, repair of cerebrospinal fluid leak, and C3 laminectomy. | No recurrence of CS during the 1-yr follow-up period. |
| Zeng Et al.  [47]  2013. | 87/M | Shortness of breath with less-than-usual daily activity, followed by the onset of CS the next day. During hospitalization, the patient had up to 10 daily CS episodes, initially presenting with facial cyanosis, fixed gaze, 10–20 s unconsciousness, and sinus bradycardia. During episodes, physical examination showed elevated jugular venous pressure, muffled heart sounds, and jugular venous distension. | Constrictive pericarditis, hypertension, Parkinson’s disease, history of smoking/Unreported. | WBC = 16.86×10⁹/L, neutrophil percentage = 88.6%. CXR showed an enlarged cardiac silhouette; chest CT imaging indicated moderate pleural effusion with pericardial thickening. Echocardiography demonstrated moderate pericardial effusion accompanied by left ventricular hypertrophy. TCD (–). ECG revealed sinus bradycardia. | Oxygen therapy, diuretics, codeine, and ceftizoxime. | Symptoms improved post-treatment. The patient was readmitted for recurrent CS 2 mo later; he and his family refused pericardiectomy. |
| Santambrogio Et al.  [48]  2008. | 72/M | Head trauma, facial contusion, and left chest wall contusion sustained during an episode of CS. The patient reported that syncope occurred following coughing, resulting in the aforementioned injuries. Similar episodes occurred 6 mo and 3 mo before admission. Dullness to percussion and diminished breath sounds over the affected hemithorax. | Colonic diverticulosis/Unreported | Blood analysis, ECG, cranial CT, echocardiography, ambulatory ECG, and carotid ultrasonography (–). CXR revealed a rib fracture and extensive opacity in the left chest wall. Chest CT imaging indicated a pleural solitary fibrous tumor. Bronchoscopy showed mild bleeding in the left upper lobe bronchus. Pathological and immunohistochemical analyses confirmed a benign pleural solitary fibrous tumor. | Surgical management included resection of the pleural solitary fibrous tumor with adjunctive wedge resection of the lingular segment. | Significant improvement in left lung function.  No recurrence of CS during 2-yr follow-up. |
| Camila, Et al.  [49]  2020. | 49/M | Dry cough, and low-grade fever for 4 wk, accompanied by rhinorrhea and conjunctival injection. Apnea and CS episodes occurred after paroxysmal severe coughing; syncope was accompanied by upward eye deviation, tonic arm stiffening with wrist flexion, and subsequent generalized limb myoclonic movements. Frequent paroxysmal coughing and CS persisted during hospitalization, at 9–12 episodes per day. | Pertussis, human immunodeficiency virus/ Unreported | WBC = 13.03×10⁹/L, lactate dehydrogenase = 232 U/L. Multiplex respiratory pathogen PCR testing (+) for *Bordetella pertussis*. Paranasal sinus CT imaging revealed diffuse mucosal thickening in all sinuses due to chronic inflammation. CXR (–). | Clarithromycin and antitussive medication | At the time of discharge, the cough had decreased in both severity and frequency. |
| Shah Et al.  [50]  2014. | 43/M | A 9-d cough with generalized fatigue, sore throat, and episodic syncope (up to 4-5 times daily, each lasting only a few seconds), accompanied by anorexia and hoarseness. On admission, paroxysmal coughing paroxysm triggered facial flushing and a 5-s generalized tonic-clonic-like seizure. Hypotension, bilateral lung wheezes, bilateral subconjunctival and periorbital subcutaneous hemorrhage, and mild edema. | Pertussis/Unreported | WBC = 8.9×10⁹/L, CRP = 21 mg/L. Serological testing confirmed *Bordetella pertussis* infection at 1 wk after admission. Chest CT imaging confirmed left basal segment atelectasis. ECG (–). | Co-trimoxazole, morphine, clarithromycin, prednisone, and nebulized therapies. | Symptoms improved at 1 wk follow-up after discharge. |
| Din Et al.  [51]  2019. | 51/M | Cough, transient syncope, mild wheezing, and a lower respiratory tract infection. The patient presented to the emergency department four times in the preceding 10 d; cough and associated syncope worsened in the pre-admission week, rendering the patient housebound. Recurrent daily CS episodes persisted during hospitalization. | Bronchial asthma, pulmonary infection, diabetes mellitus/Unreported | Blood analysis (–). CXR showed tracheal compression. Contrast-enhanced craniocervical CT imaging revealed a right retrosternal goiter causing 7-cm-long tracheal deviation and stenosis. Segmental and subsegmental branch pulmonary embolism of the right pulmonary artery. Lower extremity Doppler ultrasonography indicated left lower extremity deep vein thrombosis. | Anticoagulation with low-molecular-weight heparin and a right thyroidectomy. | At the 1-mo follow-up, the cough symptoms ameliorated, with no recurrence of CS. |
| Kubota Et al.  [52]  2024. | 73/M | The patient experienced multiple episodes of unconsciousness during coughing paroxysms, including two traffic accidents caused by syncope over the 4 yr before admission. BMI = 29.6 kg/m². | Cough-variant asthma, GERD, history of smoking./Unreported | Blood analysis, ECG, ambulatory ECG, echocardiography, EEG, carotid ultrasonography, and chest CT (–). During bedside seated Valsalva maneuver and cough provocation test with ECG monitoring, he had transient unconsciousness with absent radial pulse. Subsequent invasive arterial pressure monitoring synchronized with ECG showed a sharp systolic blood pressure drop from 110 mmHg to 50 mmHg after coughing, accompanied by unconsciousness. | Fluticasone/formoterol inhalation and vonoprazan for gastric protection | During the 3-mo follow-up, he reported no obvious discomfort. |
| Algahtani Et al.  [53]  2017. | 47/M | Cough for >10 d accompanied by CS for 2 d.  Cough, expectoration, and associated chest pain, nasal congestion, and rhinorrhea. Each syncopal episode occurred within seconds to 3 min after coughing, occasionally with brief limb convulsions, cyanosis, and hypotonia. One of the episodes resulted in a head-first fall. | Postnasal drip syndrome, Hodgkin’s lymphoma, diabetes mellitus, hypertension, hypothyroidism, coronary heart disease, pulmonary embolism/Unreported | Cranial MRI, EEG, carotid ultrasonography, ECG, 24-h ambulatory ECG monitoring, 24-h ambulatory blood pressure monitoring, echocardiography, CXR, pulmonary function tests, bronchial provocation test, and gastroscopy (–). Nasopharyngoscopy revealed mild edema of the right vocal cord and moderate laryngopharyngeal reflux disease. | Codeine, lidocaine, dextromethorphan, chlorpheniramine, levodropropizine, and morphine provided no improvement. Subsequent administration of gabapentin. | Discharged after recovery. |
| Patel Et al.  [54]  2023. | 39/F | A 5-yr history of chronic cough. Prolonged coughing episodes were associated with dyspnea. The most severe episode involved continuous coughing for 2 min, followed by a burning sensation in the head and numbness in the fingers, culminating in sudden unconsciousness. Over the past 6 mo, the frequency of syncope increased, occurring even in the absence of coughing. | Chiari malformation type I, iron deficiency anemia/Unreported | Chest CT, laryngoscopy, pulmonary function tests, bronchial provocation test, ambulatory ECG, echocardiography, EEG, and craniocervical CTA (–). MRI revealed Chiari malformation type I. | Cetirizine, intranasal fluticasone spray, esomeprazole, and albuterol did not relieve symptoms; subsequently, the patient underwent Chiari decompression, duraplasty, tonsillar resection, and C1–C2 laminectomy. | Cough resolved completely following surgery.  No CS recurrence during the 2-yr follow-up. |
| Stec Et al.  [55]  2007. | 36/F | Chronic cough accompanied by palpitations and recurrence of CS. The patient developed a cough and expectoration 9 mo before symptom onset, with persistent cough occurring every 2-3 wk alongside episodes of CS. She reported daily episodes of dry cough lasting 60–150 min. Multiple measurements indicated hypotension.  BMI = 23 kg/m², with an irregular pulse noted during coughing episodes. | PVCs, history of smoking/Unreported | Blood analysis, serum IgE, allergen testing, pulmonary function tests, CXR, bronchial hyperresponsiveness test, 24-h esophageal pH monitoring, laryngoscopy, and paranasal sinus CT (–). 24-h ambulatory ECG detected 800–2500 PVCs temporally associated with coughing episodes. Intravenous low-dose isoproterenol infusion repeatedly induced transient PVCs linked to provoked coughing. Sleep monitoring revealed thoracic movements and abrupt respiratory airflow changes consistent with coughing episodes, both coinciding with PVCs. | Verapamil, metoprolol, and propafenone were ineffective; subsequent radiofrequency ablation | Symptoms immediately ameliorated after surgery. During the 1-yr follow-up, no symptoms related to PVCs, paroxysmal cough, or syncope recurred. |
| Hu Et al.  [56]  2021. | 44/F | Cough and expectoration for 4 yr, which had been recurrently exacerbated over the 6 mo before admission. Cough and sputum production occurred primarily at night and worsened in winter. Previous treatment with antitussive medications failed to control the symptoms. The cough recurred 4 mo before admission and was accompanied by CS, with recovery of consciousness after 3 h. | PVCs/Unreported | Chest CT, pulmonary function tests, and bronchial provocation test (–). ECG showed PVCs. 24-h ambulatory ECG demonstrated that frequent PVCs were temporally associated with recurrent coughing episodes. Echocardiography revealed trace regurgitant flow. | Antiarrhythmic drug therapy proved ineffective, followed by radiofrequency ablation. | Postoperatively, the cough resolved completely, with no recurrence of arrhythmia or cough. |
| Saseedharan Et al.  [57]  2012. | 64/M | Over the 15 d before admission, the patient had a mucopurulent productive cough; over the last 2 d, 3–4 daily CS episodes developed, each lasting 30 s–1 min. One episode caused a fall with scalp contusion. A CS episode in the ICU was accompanied by a 60 mmHg systolic blood pressure drop and tachycardia.  Diminished breath sounds over the right lower chest. | Pericardial effusion, metastatic non-small cell lung cancer, hypertension, history of smoking/Unreported | WBC = 11×10⁹/L. Echocardiography showed moderate pericardial effusion with a 2-cm anechoic space posterior to the left ventricular wall; coughing induced cardiac tamponade signs including early diastolic right ventricular collapse, late diastolic right atrial invagination, and abnormal septal motion, all resolving after coughing ceased. CT imaging demonstrated concentric tracheobronchial thickening, more prominent in the right lower lobe, with distal consolidation and ground-glass opacities. Fine-needle aspiration biopsy of the right supraclavicular lymph node identified metastatic non-small cell lung cancer. Pulmonary function tests (–). | Pericardiocentesis for drainage. | After pericardiocentesis, no further episodes of CS occurred. |
| Bandyopadhyay Et al.  [58]  2014. | 43/F | A left glomus jugulare tumor and recurrence of impaired consciousness for 4 yr. Seven daily episodes on average, mainly triggered by paroxysmal coughing, presenting with fixed staring, unresponsiveness, bodily stiffness, and mild bilateral upper limb tremors, each lasting several min with occasional urinary incontinence.  Hypersomnolence, mild dysarthria, right sixth cranial nerve palsy, mildly increased lower limb muscle tone with hyperreflexia, and bilateral ankle clonus. | Left glomus jugulare tumor/Unreported | Blood analysis (–); cerebrospinal fluid pressure = 25 cmH₂O. cEEG recorded three identical episodes initiated by paroxysmal coughing in the recumbent position, followed by distal upper limb tremors lasting <1 min and mild proximal upper limb myoclonic jerks, and culminating in unconsciousness up to 10 min with hypotension and bradycardia. Ictal EEG showed diffuse synchronous and asynchronous high-amplitude 1–2 Hz delta activity. CT imaging revealed recurrent jugular paraganglioma and communicating hydrocephalus. MRI demonstrated a T2-weighted hyperintense enhancing mass at the left skull base jugular foramen, extending into the posterior fossa and below the skull base. | An external ventricular drain was inserted, with gradual resolution of somnolence and reduction in seizure frequency to once daily; a ventriculoperitoneal shunt was placed. | 2-d before discharge, she experienced one mild seizure-like episode triggered by coughing, without syncope. |
| Kusuyama Et al.  [59]  2009. | 52/M | Syncope accompanied by convulsions. The patient reported multiple episodes of vomiting following a farewell party, and, several days later, experienced sudden syncope with convulsions after a severe coughing episode. BMI = 23.23 kg/m², BP = 86/64 mmHg. | GERD/Unreported | Blood analysis, ECG, CXR, and echocardiography (–). Ambulatory ECG monitoring revealed prolonged sinus pauses following coughing episodes, accompanied by convulsive movements and unconsciousness. Each coughing episode was followed by a sinus pause and syncope. Gastroscopy showed laryngopharyngeal edema, esophageal ulceration, severe esophagitis, and acute gastric mucosal lesions. | A temporary pacemaker was implanted, and continuous lansoprazole. | Follow-up gastroscopy showed complete resolution of laryngopharyngeal edema and esophageal ulcers, with persistent reflux esophagitis.  No CS recurrence at the 6-mo follow-up. |
| Wee Et al.  [60]  2015. | 74/M | Syncope triggered by coughing, preceded by persistent shortness of breath and productive cough over several weeks. One episode of CS recurred at the time of admission. Temperature = 38.2°C, heart rate = 120 beats per min, BP = 80/60 mmHg, muffled heart sounds, and elevated jugular venous pressure. | Constrictive pericarditis, rheumatoid arthritis, gout, history of smoking/Unreported | The erythrocyte sedimentation rate 40 mm/h, and CRP = 125 mg/L. WBC (–). PCR testing and subsequent culture of pericardial tissue and pleural fluid (+) for *Mycobacterium tuberculosis*; sputum culture also grew *Mycobacterium tuberculosis*. CT imaging revealed a massive pericardial effusion and nodular opacities in the right lung apex. echocardiography confirmed the presence of a large pericardial effusion with diastolic collapse of the right ventricle. | Emergent pericardiocentesis followed by thoracoscopic pericardial window creation for drainage. Subsequent four-drug anti-tuberculosis regimen and prednisolone | Over a 2-mo follow-up, the patient’s symptoms continued to show sustained improvement. |
| Ramirez Et al.  [61]  2017. | 75/M | Worsening shortness of breath accompanied by three syncopes, two of which were associated with coughing. Each episode lasted 30 s to 1 min. BP = 151/88 mmHg, pulsus paradoxus present, grade 2/6 systolic murmur at the cardiac apex, diminished breath sounds in the lung. | Pericardial effusion,  lung adenocarcinoma, COPD, hypertension, diabetes mellitus, hyperlipidemia/Unreported | WBC = 12.8×10⁹/L; cytological examination of pericardial and pleural effusions identified adenocarcinoma. ECG showed sinus tachycardia with intraventricular conduction delay. Chest CT imaging demonstrated worsening bilateral pleural effusions and pericardial effusion. Echocardiography confirmed a large pericardial effusion with diastolic indentation and right ventricular collapse indicative of cardiac tamponade, a finding also noted on M-mode echocardiography; respiratory variation of mitral inflow E-wave velocity exceeded 25%, consistent with cardiac tamponade. | Drainage of pericardial and pleural effusions | Following treatment, the cough and dyspnea significantly improved, with no recurrence of CS. |

BMI, body mass index; BP, blood pressure; COPD, chronic obstructive pulmonary disease; CRP, C-reactive protein; CXR, chest radiography; CS, cough syncope; CT, computed tomography; CTA, computed tomography angiography; ECG, electrocardiogram; EEG, electroencephalogram; GERD, gastroesophageal reflux disease; HR, heart rate; HUTT, head-up tilt test; IgE, immunoglobulin E; MRI, magnetic resonance imaging; OSA, obstructive sleep apnea; PVCs, premature ventricular contractions; TCD, transcranial Doppler; WBC, white blood cell. d, day; h, hour; mo, month; min, minute; s, second; wk, week; yr, year; (–), negative or within the reference range; (+) positive.

**Online Resource: Video Caption**

Patient 4 developed an abrupt paroxysmal severe cough while eating and lost consciousness after >10 s, with both the patient and the bowl falling to the ground. He regained consciousness within seconds and gradually stood up.
